# A comparative analysis of sivelestat sodium hydrate and ulinastatin combination therapy in the treatment of sepsis with acute respiratory distress syndrome

**DOI:** 10.1186/s12890-024-03083-w

**Published:** 2024-06-17

**Authors:** Jian Xu, Chenfei Zhang, Keren Wu, Yanhua Qian, Wei Hu

**Affiliations:** 1 Department of Respiratory and Critical Care Medicine, Affiliated Wuxi Fifth Hospital of Jiangnan University, Wuxi, 214000 Jiangsu China; 2 Wuxi Medical College of Jiangnan University, No. 1215, Guangrui Road, Liangxi District, Wuxi, 214000 Jiangsu China; 3 Wenzhou Medical University, Wenzhou, 325035 Zhejiang China; 4 Department of Respiratory and Critical Care Medicine, the 904 Hospital of the Joint Logistics Support Force of the Chinese People’s Liberation Army, Wuxi, 214000 Jiangsu China; 5 Department of Pharmacy, the 904 Hospital of the Joint Logistics Support Force of the Chinese People’s Liberation Army, No. 101, Xingyuan Road, Liangxi District, Wuxi, 214000 Jiangsu China

**Keywords:** Sepsis with acute respiratory distress syndrome, Sivelestat sodium hydrate, Ulinastatin, Therapeutic efficacy, Respiratory function, Inflammation, Oxidative stress, Adverse reactions

## Abstract

**Objective:**

This comparative analysis aimed to investigate the efficacy of Sivelestat Sodium Hydrate (SSH) combined with Ulinastatin (UTI) in the treatment of sepsis with acute respiratory distress syndrome (ARDS).

**Methods:**

A control group and an observation group were formed with eighty-four cases of patients with sepsis with ARDS, with 42 cases in each group. The control group was intravenously injected with UTI based on conventional treatment, and the observation group was injected with SSH based on the control group. Both groups were treated continuously for 7 days, and the treatment outcomes and efficacy of both groups were observed. The Murray Lung Injury Score (MLIS), Sequential Organ Failure Assessment (SOFA), and Acute Physiology and Chronic Health Evaluation II (APACHE II) were compared. Changes in respiratory function, inflammatory factors, and oxidative stress indicators were assessed. The occurrence of adverse drug reactions was recorded.

**Results:**

The total effective rate in the observation group (95.24%) was higher than that in the control group (80.95%) (*P* < 0.05). The mechanical ventilation time, intensive care unit (ICU) hospitalization time, and duration of antimicrobial medication in the observation group were shorter and multiple organ dysfunction syndrome incidence was lower than those in the control group (*P* < 0.05). The mortality rate of patients in the observation group (35.71%) was lower than that in the control group (52.38%), but there was no statistically significant difference between the two groups (*P* > 0.05). MLIS, SOFA, and APACHE II scores in the observation group were lower than the control group (*P* < 0.05). After treatment, respiratory function, inflammation, and oxidative stress were improved in the observation group (*P* < 0.05). Adverse reactions were not significantly different between the two groups (*P* > 0.05).

**Conclusion:**

The combination of SSH plus UTI improves lung injury and pulmonary ventilation function, and reduces inflammation and oxidative stress in patients with sepsis and ARDS.

## Introduction

Sepsis, a critical organ malfunction resulting from an uncontrolled host reaction to infection, is characterized by septic shock as a form of sepsis [1]. Bacteria, fungi, or viruses can lead to sepsis, and currently, no targeted treatment exists. The primary approach is to manage the infection by controlling its source and providing antibiotics along with organ function support [2]. An immune reaction triggers significant dysfunctions in both macro and microcirculatory systems, resulting in widespread hypoperfusion and harm to various organs. As a result, individuals suffering from sepsis may exhibit malfunctions in almost all bodily systems, irrespective of the infection location [3]. Acute respiratory distress syndrome (ARDS) manifests as an acute inflammatory lung injury, marked by harm to both alveolar epithelial cells and pulmonary capillary endothelial cells [4]. Intense inflammatory reactions triggered by sepsis enhance vascular permeability, causing acute pulmonary edema and culminating in ARDS [5]. Thus, transitioning from sepsis to ARDS requires improvement in management.

Ulinastatin (UTI) known for inhibiting urinary trypsin, is recognized as a possible immunomodulator advantageous for multiple organ dysfunction syndrome by protection against organs, tissues, and endothelial cells and anti-inflammatory function [6]. UTI has provided therapeutic efficacy in the treatment of septic acute lung injury and ARDS [7, 8]. Additionally, UTI guards against acute lung damage caused by lipopolysaccharides (LPS) by enhancing inflammatory pathway activation and diminishing inflammatory mediators [9]. Neutrophil elastase (NE), crucial in the primary granules of neutrophils, is instrumental in initiating inflammation, has bactericidal effects, and shortens inflammation duration. Sivelestat Sodium Hydrate (SSH), known for inhibiting NE, shows significant promise for use in clinical settings [10]. Clinical trials indicate that SSH is effective in treating ARDS without compromising the host’s immune defense during infections [11, 12], and nonclinical trials have addressed that SSH can improve pulmonary function in severe respiratory failure [13] and symptoms of acute lung injury (ALI) [14]. In combination, this trial applied UTI and SSH in the treatment of patients with sepsis and ARDS to determine their clinical efficacy.

## Materials and methods

### Ethics statement

The study complied with the review and approval of the medical ethics committee of Affiliated Wuxi Fifth Hospital of Jiangnan University (approval number: 20,201,024). Written informed consent was acquired from all study subjects.

### Study subjects

The study subjects included 120 patients with sepsis with ARDS who were admitted to Affiliated Wuxi Fifth Hospital of Jiangnan University. Inclusion criteria: (1) Age ≥ 18 years; (2) Patient meeting the diagnostic criteria for sepsis and ARDS [15]; (3) Patients requiring mechanical ventilation; (4) Patients who are compliant and able to cooperate with the study; (5) Patients with complete clinical data. Exclusion criteria: (1) Patients with combined malignant tumors and immunodeficiency diseases; (2) Patients with allergy to the study drug; (3) Patients with a history of organ transplantation; (4) Patients in the agonal stage (death expected within 12 h).

**Fig. 1:**
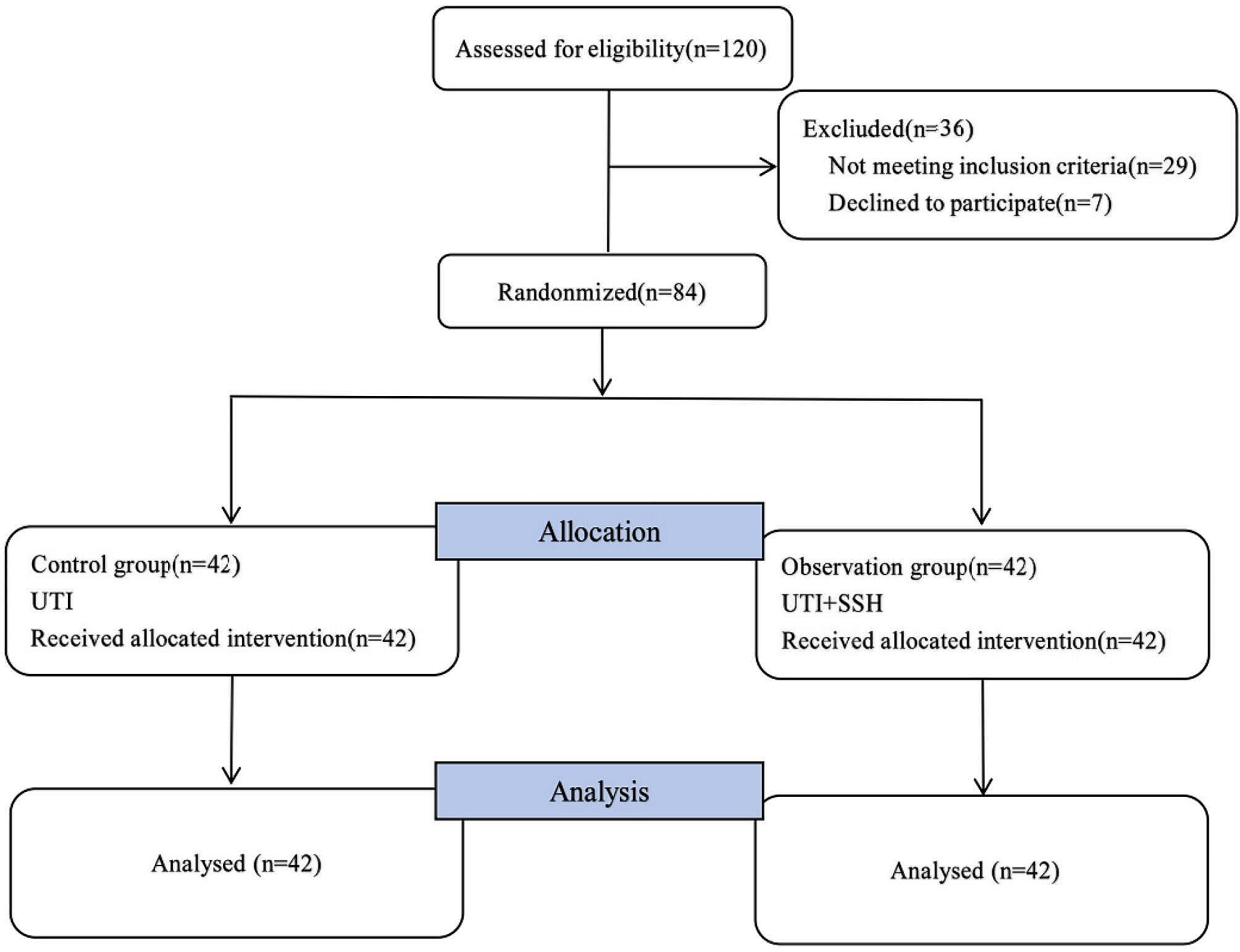
Flowchart for case enrollment

According to the inclusion and exclusion criteria, 84 patients were ultimately enrolled in this study (Fig. 1). A randomized numerical table method was employed to divide the patients into a control group and an observation group, with 42 cases in each group. The differences in general information between the two groups were not statistically significant (*P* > 0.05), as shown in Table 1.

**Table 1:** General data analysis of patients

Parameters	Control group (*n* = 42)	Observation group (*n* = 42)	*P* value
Gender (n, %)			0.824
Male	24 (57.14%)	26 (61.90%)	
Female	18 (42.86%)	16 (38.10%)	
Age (years)	58.95 ± 4.10	58.43 ± 3.95	0.553
Primary diseases (n, %)			0.88
Pulmonary infection	21 (50.00%)	23 (54.76%)	
Abdominal infection	12 (28.57%)	10 (23.81%)	
Urinary tract infection	7 (16.67%)	8 (19.05%)	
Others	2 (4.76%)	1 (2.38%)	
Basic diseases (n, %)			0.812
Hypertension	16 (38.10%)	18 (42.86%)	
Coronary heart disease	13 (30.95%)	11 (26.19%)	
Diabetes	10 (23.81%)	12 (28.57%)	
APACHE II score (points)	22.74 ± 2.02	23.12 ± 1.95	0.383

Note: APACHE II, Acute Physiology and Chronic Health Evaluation II.

### Treatments

Referring to the “Third National Consensus on Sepsis and Septic Shock” and “Guidelines for Mechanical Ventilation of Patients with Acute Respiratory Distress Syndrome (Trial)” published by the European Society of Intensive Care Med (ESICM) and the Society of Critical Care Medicine (SCCM) [16], all the patients were actively treated for the primary disease after hospitalization, including early fluid resuscitation, fluid expansion, maintenance of water-electrolyte and acid-base balance, antibacterial medication according to drug sensitivity test, mechanical ventilation, and nutritional support.

In the control group, the patients were injected with UTI (H1990134, Techpool BIO-PHARMA Co., Ltd., Guangdong, China) at 200,000 U/times, 3 times/day for 7 days consecutively, and those in the observation group were intravenously pumped with SSH (H20203093, Shanghai Huilun (Jiangsu) Pharmaceutical Co., Ltd., Shanghai, China) at 0.2 mg/kg/h. The dosage was determined according to the patient’s body mass. Continuous treatment was carried out for 7 days, and the treatment program was adjusted according to the patient’s specific recovery situation.

### Clinical effect

Clinical effect was observed regarding the “Clinical Disease Diagnosis Criteria for Cure and Improvement”. Obviously effective: Body temperature returns to normal, respiratory rate < 20 breaths/min, heart rate < 90 beats/min, white blood cell (WBC) count (4–10) × 10^9^/L; Effective: The condition improves significantly, but the body temperature, respiratory rate, heart rate, and WBC count fall short of cure standards; Ineffective: The condition improves, and the body temperature, respiratory rate, and heart rate improve, but not significantly, or the condition deteriorates. Total effective rate = number of (obviously effective + effective)/total number of cases.

### Short-term prognosis

Mechanical ventilation time, ICU hospitalization, and duration of antimicrobial medication were recorded. The incidence of multiple organ dysfunction syndrome (MODS) during treatment and mortality rate within 28 days of admission were calculated.

### Lung injury and prognostic scores

Murray Lung Injury Score (MLIS), Sequential Organ Failure Assessment (SOFA), and Acute Physiology and Chronic Health Evaluation II (APACHE II) were evaluated before and after 7 days of treatment, respectively. MLIS was evaluated from four aspects, namely, chest X-ray, positive end-expiratory pressure, hypoxemia, and respiratory compliance, with a total score of 0–4. The higher the score, the more severe the lung injury. SOFA was assessed from respiratory, coagulation, hepatic, cardiovascular, central nervous, and renal systems, with a total score of 0–24. The SOFA score was positively correlated with the severity of the disease. APACHE II consists of three components: acute physiology score, age score, and chronic health score, with a maximum score of 71. The higher the total scores, the worse the prognosis.

### Respiratory function

Arterial blood partial pressure of oxygen (PaO_2_), partial pressure of carbon dioxide (PaCO_2_) and oxygenation index (PaO_2_/FiO_2_) were measured using an arterial blood gas analyzer, PL2000PLUS (Redumit Medical Equipment (Shanghai) Co., Ltd., Shanghai, China) before and after 7 days of treatment, respectively.

### Inflammatory factors and oxidative stress indicators

Fasting peripheral venous blood was acquired early in the morning from both groups before and after 7 days of treatment, respectively, and the serum was centrifuged and separated. WBC count was determined by automatic hematology analyzer, and procalcitonin (PCT), C-reactive protein (CRP), and interleukin-6 (IL-6) were measured by enzyme-linked immunosorbent assay (ELISA). Superoxide dismutase (SOD) was measured by xanthine assay, malondialdehyde (MDA) by thiobarbituric colorimetric assay, and glutathione peroxidase (GSH-Px) by ELISA.

### Incidence of adverse reactions

Adverse reactions were recorded, including nausea and vomiting, rash and itching, liver and kidney dysfunction, platelet abnormality, and hematocytopenia. The incidence of adverse reactions was compared.

### Statistical analysis

SPSS 22.0 software (IBM, NY, USA) and GraphPad Prism 6.0 software (Graph Pad Inc., CA, USA) were applied to process the experimental data. Measurement data (expressed as mean ± standard deviation) showed normal distribution and homogeneity of variance. Paired t-test was used for measurement data before and after treatment, and independent sample t-test was utilized for individual comparisons between groups. Enumeration data (expressed as %) were analyzed by χ^2^ test. The test level was α = 0.05, and *P* < 0.05 was regarded as a statistically significant difference.

## Results

### Clinical effect

The total effective rate of treatment in the observation group (95.24%) was higher than that in the control group (80.95%) (*P* < 0.05). Overall, treatment with SSH combined with UTI was more effective in sepsis with ARDS patients (Table 2).

**Table 2:** Comparison of efficacy between the two groups

Groups	Obviously effective	Effective	Ineffective	Total effective rate
Control group (*n* = 42)	25 (59.52%)	9 (21.43%)	8 (19.05%)	34 (80.95%)
Observation group (*n* = 42)	34 (80.95%)	6 (14.29%)	2 (4.76%)	40 (95.24%)
*P* value				0.018

### Short-term prognosis

The mechanical ventilation time, ICU hospitalization, and duration of antimicrobial medication in the observation group were shorter than those in the control group, and MODS incidence was lower than those in the control group (*P* < 0.05). The mortality rate of patients in the observation group (35.71%) was lower than that in the control group (52.38%), but there was no statistically significant difference between the two groups (*P* > 0.05). Patients with sepsis with ARDS treated with SSH combined with UTI have a better short-term prognosis (Table 3).

**Table 3:** Comparison of short-term prognosis between the two groups

Groups	Mechanical ventilation time (days)	ICU hospitalization (days)	Duration of antimicrobial medication (days)	MODS incidence	Mortality rate
Control group (*n* = 42)	9.07 ± 1.09	11.36 ± 1.95	10.36 ± 1.64	20 (47.62%)	22 (52.38%)
Observation group (*n* = 42)	7.12 ± 0.59	9.19 ± 1.25	8.40 ± 1.11	11 (26.19%)	15 (35.71%)
*P* value	< 0.001	< 0.001	< 0.001	0.042	0.124

Note: MODS, multiple organ dysfunction syndrome

### MLIS, SOFA, and APACHE II scores

Before treatment, the differences were not statistically significant in MLIS, SOFA, and APACHE II scores (*P* > 0.05). After 7 days of treatment, MLIS, SOFA, and APACHE II scores of both groups were lower than before treatment, and the decrease was greater in the observation group than the control group (*P* < 0.05). A combination of SSH and UTI may improve lung injury and prognosis in patients with sepsis with ARDS (Table 4).

**Table 4:** Comparison of MLIS, SOFA, and APACHE II scores between the two groups before and after treatment

Groups	MLIS score (points)		SOFA score (points)		APACHEII score (points)	
	Before treatment	7 days after treatment	Before treatment	7 days after treatment	Before treatment	7 days after treatment
Control group (*n* = 42)	2.65 ± 0.50	1.39 ± 0.36^*^	9.14 ± 1.24	5.24 ± 0.88^*^	22.74 ± 2.02	15.38 ± 1.13^*^
Observation group (*n* = 42)	2.63 ± 0.48	0.75 ± 0.14^*^	9.12 ± 1.15	4.02 ± 0.60^*^	23.12 ± 1.95	12.74 ± 0.96^*^
*P* value	0.806	< 0.001	0.928	< 0.001	0.383	< 0.001

Note: Compared with before treatment, * *P* < 0.05. MLIS, Murray Lung Injury Score; SOFA, Sequential Organ Failure Assessment; APACHE II, Acute Physiology and Chronic Health Evaluation II.

### Respiratory function

Neither group had statistically significant differences in PaO_2_, PaCO_2_, or PaO_2_/FiO_2_ before treatment (*P* > 0.05). After 7 days of treatment, PaO_2_ and PaO_2_/FiO_2_ of both groups were higher while PaCO_2_ was lower than before treatment. The change was more significant in the observation group (*P* < 0.05). Combined treatment of SSH and UTI improves lung ventilation function in patients with sepsis and ARDS (Table 5).

**Table 5:** Comparison of respiratory function indices before and after treatment between the two groups

Groups	PaO_2_ (mmHg)		PaCO_2_ (mmHg)		PaO_2_/FiO_2_	
	Before treatment	7 days after treatment	Before treatment	7 days after treatment	Before treatment	7 days after treatment
Control group (*n* = 42)	51.38 ± 5.29	74.14 ± 8.04^*^	58.29 ± 6.11	46.24 ± 5.09^*^	78.16 ± 8.39	183.54 ± 21.74^*^
Observation group (*n* = 42)	52.74 ± 5.84	86.57 ± 9.18^*^	58.14 ± 6.28	40.12 ± 4.37^*^	76.92 ± 8.85	235.76 ± 29.83^*^
*P* value	0.268	< 0.001	0.916	< 0.001	0.512	< 0.001

Note: Compared with before treatment, * *P* < 0.05

### Inflammatory factors and oxidative stress indices

Inflammatory factors and oxidative stress indicators were not statistically significantly different between the two groups before treatment (*P* > 0.05). After treatment, WBC, PCT, CRP, IL-6, and MDA of both groups decreased, and SOD and GSH-Px increased. The range of alternations in these indices was more obvious in the observation group (*P* < 0.05). Patients with sepsis with ARDS benefit from treatment with SSH + UTI for reducing systemic inflammation and oxidative stress (Table 6).

**Table 6:** Comparison of inflammatory factors and oxidative stress indices between the two groups before and after treatment

Parameters	Time	Control group (*n* = 42)	Observation group (*n* = 42)	*P* value
WBC (×10^9^/L)	Before treatment	14.35 ± 3.92	14.19 ± 3.82	0.851
	7 days after treatment	11.83 ± 2.47^*^	9.04 ± 2.15^*^	< 0.001
PCT (mg/mL)	Before treatment	29.84 ± 4.26	30.05 ± 4.41	0.824
	7 days after treatment	16.49 ± 3.28^*^	11.24 ± 2.71^*^	< 0.001
CRP (mg/L)	Before treatment	78.96 ± 8.51	78.13 ± 8.44	0.655
	7 days after treatment	38.49 ± 5.51^*^	20.63 ± 4.02^*^	< 0.001
IL-6 (pg/mL)	Before treatment	295.76 ± 35.49	301.75 ± 34.92	0.438
	7 days after treatment	201.85 ± 27.53^*^	130.18 ± 20.73^*^	< 0.001
MDA (nmol/L)	Before treatment	9.84 ± 1.28	9.71 ± 1.16	0.627
	7 days after treatment	7.25 ± 0.93^*^	5.53 ± 0.71^*^	< 0.001
SOD (U/L)	Before treatment	12.08 ± 2.01	12.16 ± 1.93	0.853
	7 days after treatment	19.96 ± 3.25^*^	28.04 ± 3.94^*^	< 0.001
GSH-Px (g/L)	Before treatment	41.28 ± 5.69	40.95 ± 5.71	0.792
	7 days after treatment	53.67 ± 6.28^*^	69.93 ± 7.08^*^	< 0.001

Note: Compared with before treatment, * *P* < 0.05. WBC, white blood cells; PCT, procalcitonin; CRP, C-reactive protein; IL-6, interleukin-6; MDA, malondialdehyde; SOD, superoxide dismutase; GSH-Px, glutathione peroxidase

### Adverse reactions

Adverse reactions are not significantly different between the two groups of patients before treatment (*P* > 0.05). Combining SSH with UTI shows superior results to monotherapy with a high degree of safety (Table 7).

**Table 7:** Comparison of the incidence of adverse reactions between the two groups

Groups	Nausea and vomiting	Rash and itching	Liver and kidney dysfunction	Blood platelet disorder	Hemocytopenia	Incidence
Control group (*n* = 42)	2 (4.76%)	2 (4.76%)	0 (0.00%)	0 (0.00%)	1 (2.38%)	5 (11.90%)
Observation group (*n* = 42)	4 (9.52%)	1 (2.38%)	1 (2.38%)	1 (2.38%)	1 (2.38%)	8 (19.05%)
*P* value						0.366

## Discussion

ARDS is a common complication of sepsis, and septic patients with ARDS are at a high mortality risk of 20–50% [17]. UTI is a protease inhibitor, which is mainly secreted by the liver. Its main mechanism of action is to balance the proportion of T lymphocytes, improve the activity of SOD, inhibit the spread of inflammatory factors, and then control systemic inflammation [18]. SSH selectively inhibits NE activity, thereby attenuating lung tissue damage and inflammatory response [19]. However, its combination with UTI has not been completely discussed yet. Thus, in the current study, patients suffering from sepsis and ARDS received either a single UTI treatment or a combination of UTI and SSH therapy, with this study further validating the effectiveness of the combined approach.

According to previous reports, SSH has been believed to be a therapeutic candidate for ARDS associated with sepsis. For instance, after surgery for abdominal sepsis, patients with ARDS who received SSH experienced early improvements in their multiple organ dysfunction score and oxygenation, as well as early ventilator weaning and release from the ICU [20]. Also, SSH can decrease the need for mechanical ventilation and ICU hospitalization and raise oxygenation in patients with septic ARDS [21]. Furthermore, suppression of NE by SSH reduced lung injury through suppression of the inflammatory signaling cascade in rats [22]. The observation group showed a higher total effective rate of treatment, shortened mechanical ventilation time, ICU hospitalization, and duration of antibacterial medication, and lower MODS incidence and mortality rate. These data indicated that UTI and SSH combined therapy improved the clinical outcome and short-term prognosis of patients with sepsis and ARDS. Further, compared with the two groups of patients, SSH could reduce the MLIS score, APACHE II score, and SOFA score of patients within the same treatment time window, that is, improve the severity of patients, with a view to obtaining better clinical outcomes. In addition, elevated PaO_2_ and PaO_2_/FiO_2_ and reduced PaCO_2_ were detected in the observation group compared to the control group, suggesting that SSH combined with UTI can improve lung function.

As a physiological response to foreign organisms, inflammation consists of a multitude of physiological reactions. One of the underlying causes of inflammation is an imbalance between natural antioxidants in the body [23]. The current trial detected lower WBC, PCT, CRP, IL-6, and MDA and higher SOD and GSH-Px in the observation group in comparison to the control group. It is suggested that the treatment of SSH combined with UTI is beneficial in reducing the inflammatory response and oxidative stress of the human body. The research further revealed no difference in the incidence of adverse events between the groups during treatment, suggesting that combining SSH and UTI did not escalate patient adverse reactions and was deemed safe.

The specific mechanism of SSH in the treatment of sepsis with ARDS may be that NE is over-activated, which improves the permeability of pulmonary blood vessels, tracheal contraction, and secretion of inflammatory mediators, and induces ARDS. After treatment with SSH, it can inhibit NE activity as a specific and highly effective NE inhibitor. At the same time, the lung protection effect can be exerted through multiple ways, such as pro-inflammatory cytokines in alveolar lavage fluid are significantly decreased, and the clearance rate of bacteria is increased.

In summary, the combination of SSH and UTI is more effective in patients with sepsis associated with ARDS, improves lung injury and ventilation, and reduces systemic inflammation and oxidative stress response. This paper reveals that the combination of SSH and UTI is effective in the clinical treatment of sepsis associated with ARDS. Nevertheless, the calculation of sample size was not carried out and the univariate and multivariate analyses of the influencing factors of the prognosis of ARDS were not performed in our study, which are the main limitations for our study. Therefore, further study is necessary to validate our results.

## Data Availability

The datasets used and/or analyzed during the current study available from the corresponding author on reasonable request.
